# A Multidisciplinary Atrial Fibrillation Clinic 

**DOI:** 10.2174/157340313805076287

**Published:** 2013-02

**Authors:** Huyentran N Tran, Javad Tafreshi, Elvin A Hernandez, Sudha M Pai, Vilma I Torres, Ramdas G Pai

**Affiliations:** 1 Loma Linda University School of Pharmacy and Loma Linda University Medical Center; 2 Loma Linda University School of Medicine and Loma Linda University Medical Center

**Keywords:** Atrial fibrillation clinic.

## Abstract

**Background:**

Reports in the literature indicate that specialty clinics focusing on management of patients with specific chronic disorders have a significant positive impact on patient outcomes. Atrial fibrillation (AF), one of the most common forms of cardiac arrhythmia, affects millions of patients. Outcome data regarding the impact of managing patients with AF are limited. We established a specialty clinic focusing on management of patients with AF. The objective of our study was to evaluate the outcomes of treating AF patients in this clinic.

**Methods:**

A team consisting of electrophysiologists and pharmacists designed a specific plan for managing and educating patients. This plan consisted of evaluation, implementation of an individualized treatment plan, patient education, medication management, and follow-up care. We reviewed the outcomes of patients who had clinic visits between November 2011 and March 2012. The primary outcome was the incidence of AF-related hospitalizations and stroke.

**Results:**

Seventy one patients were included in the analysis. Out of 71 patients, we identified 17 (23.9%) patients who were hospitalized. Two of these 17 hospitalized patients had ischemic stroke events.

**Conclusion:**

When compared to published data in the existing literature, managing AF patients in specialty clinics reduces the incidence of AF-related hospitalizations and stroke.

## BACKGROUND

Atrial fibrillation (AF) is one of the most common forms of cardiac arrhythmia and is known to increase the risk of stroke by several folds [1]. The prevalence of AF in the general population is about 0.4% to 1% and increases up to 8% in patients over the age of 80 [2]. Approximately one-third of hospital admissions is due to AF [3]. The number of hospitalizations from AF has increased by 66% over the last 20 years, mostly because of earlier diagnosis, the aging population, and increasing number of patients with heart disease [4]. Treatment of patients with AF includes controlling heart rate, converting to and maintaining sinus rhythm, and prevention of thromboembolic events. Non-adherence to evidence-based guidelines is a common and serious problem in the management of AF and is associated with increased morbidity and mortality. [5] Reports in the literature indicate that specialty clinics may have a significant positive impact on patient outcomes [6-13]. Although there are few published reports in the literature, these reports usually do not indicate the potential economic and clinical impacts of managing AF patients [5-12]. The objective of this study was to evaluate the impact of an individualized and focused effort on outcomes associated with the management AF patients in an ambulatory care setting. We conducted our study in a large, tertiary care facility. This facility consists of a large hospital, children’s hospital, and clinics, including an ambulatory care cardiology clinic. The primary outcome was the incidence of AF-related hospitalizations and strokes. Percent time in international normalized ratio (INR) therapeutic range (TTR) was evaluated as a secondary outcome.

## STUDY HYPOTHESIS

We hypothesized that an individualized, focused approach would result in more positive clinical and economical outcomes for AF-related hospitalizations and strokes when compared to the traditional models of care. 

## METHODS

A team consisting of electrophysiologists and pharmacists designed a specific and detailed plan for managing and educating patients. Evaluation and treatment were designed to address the needs of patients by specially-trained clinicians to achieve an optimal and individualized care for each patient. Care consisted of evaluation, implementation of an individualized treatment plan, patient education, medication management, and follow-up care. Extensive patient education included providing treatment options, anticoagulation therapy, dietary instructions, rate and rhythm control strategies, intervention options, dose titration, treatment of AF-associated risk factors, and management or prevention of common adverse drug effects. Patient education and management was based on treatment protocols (Flow diagrams 1-5). The most current American College of Cardiology Foundation/American Heart Association AF Practice Guidelines were the primary source for developing the protocol. Patients were scheduled for routine follow-up visits in 1 month, 3 months, and 6 months, or as medically warranted. Frequency of visits varied based on patient characteristics and medications used.

Clinicians contacted the patients by phone when needed and these encounters were documented. The importance of lifestyle modifications (including smoking cessation, healthy diet, and exercise) were emphasized during patient encounters. In collaboration with electrophysiologists, pharmacists participated in patient clinic visits, shared clinical responsibilities, and documented each patient encounter in the medical records. Documentation included initial AF clinic referral consults, baseline assessments, physical assessments, laboratory results, treatment plans, instructions, progress notes, follow-up visits, and phone encounters. All prescriptions for AF or ancillary medications were documented during each clinic visit. Patients referred to the AF clinic continued to be seen in the clinic on a scheduled basis. Patients were discharged from the clinic if circumstances did not allow them to be followed on a regular basis. We reviewed the records of patients who had clinic visits between November 01, 2011 and March 31, 2012. Patients referred to the AF clinic were enrolled in the study using the following eligibility criteria: (1) established diagnosis of AF and (2) age > 18 years. Patients were excluded from the study if they were newly admitted to the clinic or if they had INR values of 1.2 or below. We excluded patients who had missing data or patients with no follow up visits. The study was approved by the Institutional Review Board of the sponsoring organization. 

## DATA COLLECTION

Data collected from medical records included medical history, medication profile, clinical laboratory values, and demographics. The incidence of hospitalizations and strokes were documented on data collection forms and a composite spreadsheet. 

## STATISTICAL ANALYSIS

Cardiovascular hospitalizations consisted of hospitalization due to heart failure, underlying arrhythmia (AF, tachycardia, palpitation, etc.), stroke due to AF, and acute myocardial infarction. The primary outcome variable was defined as a binary dependent variable representing whether or not a patient was hospitalized for cardiovascular problems. We identified demographic and clinical independent variables that were measured on a continuous scale such as age in years, total cholesterol, high density lipoprotein (HDL), low density lipoprotein (LDL), triglycerides, or body mass index (BMI). Nominal categorical variables were also collected such as gender, ethnicity, medication use (whether or not medications such as warfarin, dabigatran, or rivaroxaban were used), or past medical history (whether or not patients had a past medical history of specific disease such as hypertension, coronary artery disease, or heart failure). Patient demographic and clinical characteristics were compared between AF patients who were not hospitalized or hospitalized due to non-cardiac issues to patients who were hospitalized from cardiac problems. We used the independent samples t-test for continuous level variables and the Mann-Whitney U rank-sum test for data that met the criteria for nonparametric analyses. For categorical variables, we conducted X^2^ tests for independence and Cochrane Mantel Haenszel tests, where appropriate. Alpha levels of significance were set at two-sided 0.05 to determine statistical significance. We conducted a univariable and bivariable analyses to describe our study population in relation to our primary outcome variable of whether or not a patient was hospitalized for cardiac problem(s) based on demographic characteristics such as age in years, gender, and ethnicity. The bivariable analysis also included comparing clinical independent variables in relation to whether or not patients had cardiovascular hospitalizations. We then conducted a multivariable analysis which consisted of identifying any statistically significant associations between the independent variables and the primary dependent variables. Statistically significant associations between such variables or those variables which have been identified through past literature were further analyzed using logistic regression. The results of the logistic regression were used to identify an appropriate model of clinical or demographic predictors of the primary dependent variable of whether or not patients were hospitalized with cardiac origin. To ensure adequate power to detect statistical significant effects in the multivariable analysis, we used a minimum number of events per variable (EPV) technique [14]. With the EPV technique, the dependent variable’s number of positive events determined the number of independent variables that may be used in the logistic regression model, where for every 10 events of a dependent variable, one independent variable or covariate may be used in the model [14]. Each *positive event* was considered to be an occurrence of a particular outcome under investigation. In our study, cardiovascular hospitalization was considered as a positive event. All of our analyses were conducted with SPSS Version 20.0 (SPSS Inc., Chicago, Illinois).

## RESULTS

We reviewed the profiles of a total of 106 patients with AF who had clinic visits between November 01, 2011 and March 31, 2012. Based on our eligibility requirements, 71 patients were included in the study analysis and their patient medical records were examined retrospectively for the previous two years. Out of 71 patients, we identified 17 (23.9%) patients who were hospitalized. Two of these 17 hospitalized patients had ischemic stroke events. Of these two patients with ischemic stroke, one patient was on an oral anticoagulant with a TTR of 73%. Table **1** represents the results from the univariable and bivariable analyses of the hospitalized and non-hospitalized patients. There were no statistically significant differences between the two AF groups regarding mean age in years, gender, and ethnicity. Although past medical history for both hypertension and coronary artery disease appeared to be lower for patients hospitalized when compared to non-hospitalized patients, there was no significant difference between the two groups. When examining warfarin and dabigatran treatments (Table **1**), hospitalized AF patients appeared to have lower frequencies of being treated with warfarin (21.7%) and dabigatran (12.5%) when compared to non-hospitalized patients, although not statistically significant. Mean laboratory results, such as total cholesterol, HDL, LDL, triglycerides, aspartate aminotransferase (AST) level, alanine transaminase (ALT) level, thyroid stimulating hormone (TSH) level, mean TTR results, and free T4 level, were not statistically different between the two AF groups. However, the mean number of clinic visits for hospitalized AF patients were significantly higher than non-hospitalized patients (Table **1**). 

In terms of the multivariable analysis, we constructed several logistic regression models using the EPV technique. We conservatively constructed regression models with AF hospitalization as the dependent variable regressed on one independent variable, in order to maximize power using the EPV method. In separate models, categorical covariates such as gender, ethnicity, past medical histories of hypertension, coronary artery disease, and heart failure, age, and BMI, were found to have odds ratios that were not statistically significant (Table **2**).

## DISCUSSION

AF is the most common significant arrhythmia in the United States, affecting more than 2.5 million adults with an increasing prevalence with age [2]. By 2050, the prevalence of AF is projected to increase to 5.6 million adults and approximately 90% of the patients will be 65 years and older [15]. AF is associated with a five-fold increase in the risk of stroke [16], increased risk of cardiovascular hospitalization [17], and doubling the risk of all-cause mortality [16]. Furthermore, cardiac complications in patients with AF are more common and costly compared to patients without AF [18]. For example, within the first year following an AF diagnosis, patients with AF were more likely to have heart failure, stroke, chest pain, tachycardia, palpitations or acute MI than those without the disease. As a result, hospitalizations related to AF have been increasing. Medical costs are generally five-fold higher in patients with AF than in those without the disease [19]. In addition, stroke is the most common and devastating complication of AF [16, 20, 21]. The percentage of strokes attributed to AF increases steeply with aging from 1.5% at 50-59 years of age to 23.5% at 80-89 years of age [1, 22, 23]. According to the Healthcare Cost and Utilization Project published in 2005, the mean length of stay per ischemic stroke was 5.6 days [24]. With a mean cost per day at $1,600, the cost approaches $9,000 per hospitalization per patient [24]. Moreover, the quality of life in these patients is markedly affected due to poor mental health and limited physical and social functioning [25]. According to stroke statistics in 2008, the mean lifetime cost of ischemic stroke in the United States is estimated to be $140,000 per person. This includes inpatient, rehabilitation, and follow-up care costs [26]. With an increasing prevalence and costly complications, AF imposes a huge and growing economic burden on health care systems. According to a study published in 2006, researchers estimated the total medical cost for treatment of AF patients to be $6.65 billion [27]. In 2008, Medicare alone paid more than twice that number, $15.7 billion annually, to treat patients newly diagnosed with AF [18]. Hospitalizations account for the largest share of that expense [18]. 

Our study examined the potential benefits of an individualized and focused care for patients with AF. Our goal was to limit hospital utilizations and inpatient costs by keeping medical costs within the outpatient setting. 

At our clinic, we had 17 hospitalizations. This accounted for a 23.9% admission rate occurring within 1 year compared to a 65.8% admission rate nationally occurring within 6 months, as reported in literature [28]. Regarding the number of AF-related strokes, our study identified only two cases, both of which occurred in patients above 80 years of age. In our study, these two ischemic strokes accounted for a rate of 2.82%. This figure was much lower than the estimated rate of 23.5% per year published in the literature [1, 22, 23]. 

Based on our preliminary data, our study had a potential estimated savings of $2,000,000 in life-time cost of stroke and $125,000 in hospital stay. Furthermore, our mean TTR frequency of 87.4% was markedly higher than the traditional models with an average TTR of 62.9% [29]. Compared to published national data, our hospitalization and stroke rates were lower, with a resulting potentially significant cost savings. We attribute this finding to meeting the specific needs of AF patients by paying a particular attention to their education, treatment, and follow-up. 

## LIMITATIONS

Our results indicate that several traditional covariates that are common in patients with AF, such as past medical history of hypertension, heart failure, coronary artery disease, age, BMI, gender, or ethnicity were not statistically associated with hospitalization with AF. These results may be limited due to the sample size of the hospitalized AF patients. Further research should focus on increasing the number of patients willing to participate in such studies in order to increase statistical power to detect effects. Our results may also be limited from generalization to the larger population in the United States because of the relatively low number of patients evaluated in this study.

## CONCLUSIONS

Outcomes in AF patients can be improved by an individualized and focused approach to care. AF management consisting of evaluation, implementation of an individualized treatment plan, patient education, medication management, and follow ups may improve outcomes and decrease costs.

## Figures and Tables

**Flow diagram (1):**
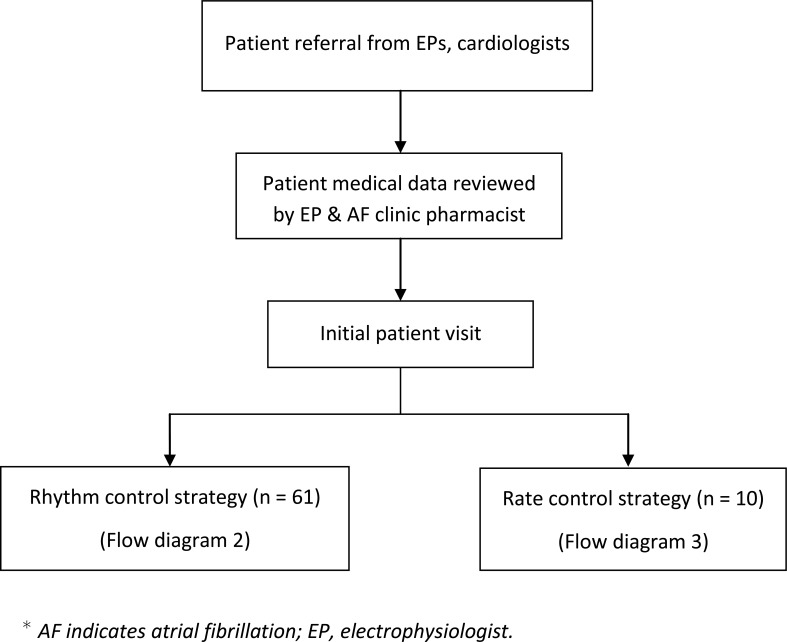
AF Care Pathway in an Ambulatory Care Setting.

**Flow diagram (2):**
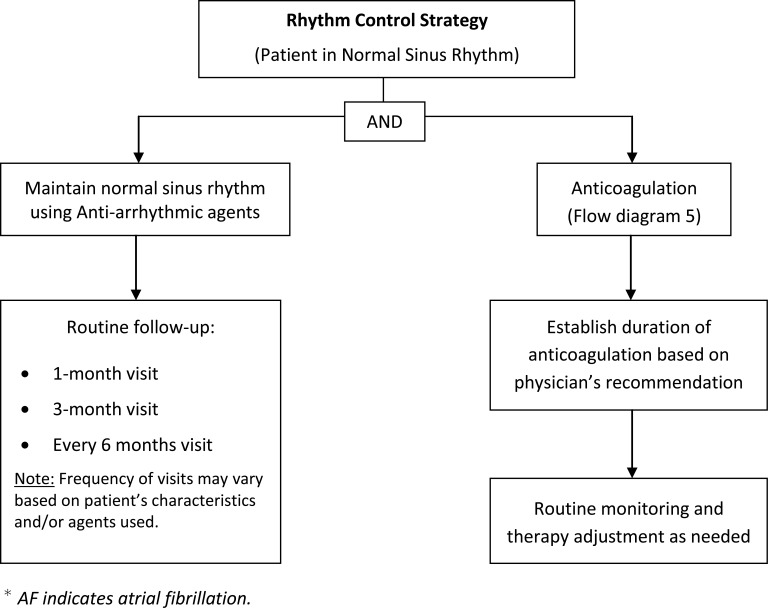
Rhythm-Control Strategy in AF.

**Flow diagram (3):**
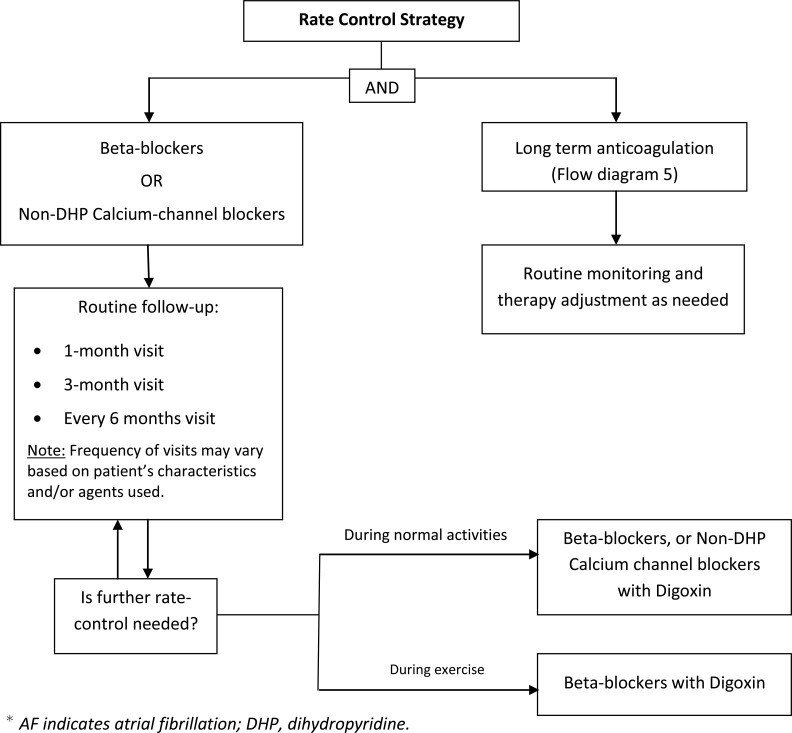
Rate-Control in Patients with Rapid AF.

**Flow diagram (4):**
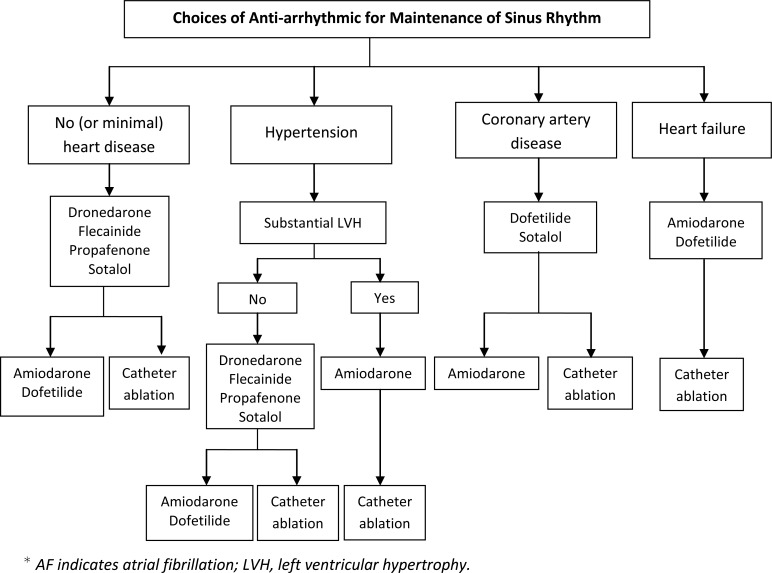
Rhythm-control with Recurrent Paroxysmal or Persistent AF.

**Flow diagram (5):**
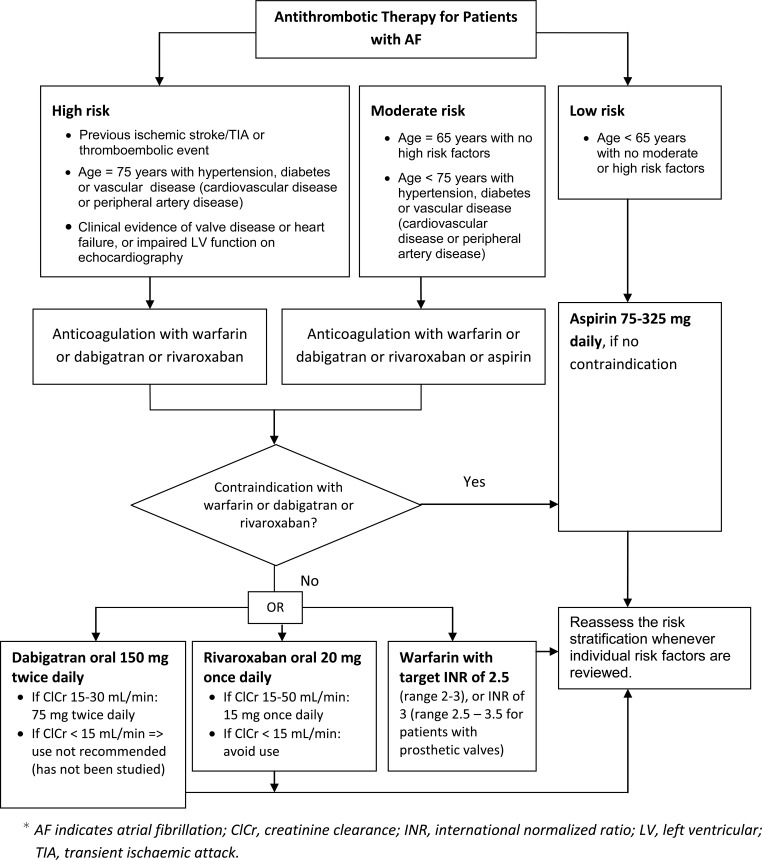
Stroke risk stratification and thromboprophylaxis.

**Table 1.** Demographics and Description Cohort Information (N=71) Categorized by Atrial Fibrillation Patients without Hospitalization and Patients with Hospitalization

Demographic Variable	Non-hospitalized Atrial Fibrillation (n=54)	Hospitalized Atrial Fibrillation (n=17)	p-value (α=0.05)
Mean age years (SD)	71.7 (9.54)	72.0 (11.8)	0.911
Gender (%)			0.443
Male	39.4	9.9	
Female	36.6	14.1	
Ethnicity (%)			0.329
Caucasian	53.5	16.9	
African American	2.8	1.4	
Hispanic/Latino	12.7	2.8	
Asian/Pacific Islander	1.4	2.8	
Other	5.6	0	
Mean Body Mass Index (SD)	31.2 (7.013)	27.6 (3.94)	0.071
Past Medical History (%)			
Hypertension	81.1	18.9	0.406
Coronary Artery Disease	72.7	27.3	0.704
Educational Intervention received (%)	72.1	27.9	0.232
TTR (avg. %)	89.2 (16.04)	80.8 (20.11)	0.196
Warfarin Treatment, n (%)	36 (78.3)	10 (21.7)	0.349
Dabigatran Treatment, n (%)	7 (87.5)	1 (12.5)	0.648
Rivaroxaban Treatment, n (%)	1 (50.0)	1 (50.0)	0.767
Mean number of Warfarin dose changes (SD)	0.94 (1.605)	1.70 (1.252)	0.177
Mean number of clinic visits (SD)	1.78 (0.963)	2.65 (1.935)	0.018*
Mean Laboratory Results (SD)			
Cholesterol	153.0 (27.50)	173.3 (37.03)	0.069
HDL	54.8 (14.50)	51.9 (16.95)	0.600
LDL	91.1 (27.48)	111.8 (40.06)	0.074
Triglycerides	90.8 (49.57)	114.4 (52.45)	0.192
AST	22.9 (6.99)	22.5 (7.67)	0.863
ALT	22.0 (10.42)	19.5 (6.30)	0.388
TSH	2.8 (1.90)	3.2 (1.43)	0.553
Free T4	2.2 (2.13)	3.6 (3.80)	0.220

* = alpha level of significance < 0.05

AST indicates aspartate aminotransferase; ALT, alanine transaminase; HDL, high density lipid; LDL, low density lipid; SD, standard deviation; TSH, thyroid-stimulating hormone; TTR, percent time in international normalized ratio therapeutic range.

**Table 2.** Multivariable Analysis with AF Hospitalization as the Dependent Variable Regressed Separately on Various Covariates

Covariate	Odds ratio	95% CI	p-value
Gender	0.650	0.216-1.960	0.444
Ethnicity	1.055	0.662-1.680	0.822
PMH - Hypertension	1.607	0.522-4.946	0.408
PMH - Coronary Artery Disease	0.754	0.175-3.254	0.705
PMH - Heart Failure	3.326	0.392-28.239	0.271
Age (years)	0.997	0.944-1.053	0.910
BMI	1.105	0.989-1.234	0.077

* AF indicates atrial fibrillation; BMI, body mass index; PMH, past medical history.
